# Wavelet clustering analysis as a tool for characterizing community structure in the human microbiome

**DOI:** 10.1038/s41598-023-34713-8

**Published:** 2023-05-17

**Authors:** Elisa Benincà, Susanne Pinto, Bernard Cazelles, Susana Fuentes, Sudarshan Shetty, Johannes A. Bogaards

**Affiliations:** 1 Centre for Infectious Disease Control, National Institute for Public Health and the Environment (RIVM), Bilthoven, The Netherlands; 2 Biomedical Data Sciences, Leiden UMC, Leiden, The Netherlands; 3 CNRS UMR-8197, IBENS, Ecole Normale Supérieure, Paris, France; 4 Sorbonne Université, UMMISCO, Paris, France; 5 Department of Medical Microbiology and Infection Prevention, UMC Groningen, Groningen, The Netherlands; 6 Department of Epidemiology & Data Science, Amsterdam UMC location VUMC, Amsterdam, The Netherlands; 7 Amsterdam Institute for Infection and Immunity, Amsterdam UMC, Amsterdam, The Netherlands

**Keywords:** Community ecology, Microbial ecology, Microbiome, Population dynamics

## Abstract

Human microbiome research is helped by the characterization of microbial networks, as these may reveal key microbes that can be targeted for beneficial health effects. Prevailing methods of microbial network characterization are based on measures of association, often applied to limited sampling points in time. Here, we demonstrate the potential of wavelet clustering, a technique that clusters time series based on similarities in their spectral characteristics. We illustrate this technique with synthetic time series and apply wavelet clustering to densely sampled human gut microbiome time series. We compare our results with hierarchical clustering based on temporal correlations in abundance, within and across individuals, and show that the cluster trees obtained by using either method are significantly different in terms of elements clustered together, branching structure and total branch length. By capitalizing on the dynamic nature of the human microbiome, wavelet clustering reveals community structures that remain obscured in correlation-based methods.

## Introduction

The human microbiome is the collective of microbial communities living on the various surfaces of the human body. These communities consist of microorganisms which do not live in isolation but interact with each other and with their human host^1,2^. In the past decade, thanks to advances in sequencing techniques and data analysis, an increasing number of studies have attempted to gain ecological insights from microbiome abundance data, e.g. by reconstructing networks of interacting species with the nodes representing the microorganisms and the edges representing the dependencies between them^3^.

Most of the studies that aim to reconstruct the network of interacting species are based on measures of co-occurrence, e.g. using correlations between pairs of species as proxies of between-species dependencies^4–6^. Despite the popularity of such methods in microbiome studies^5,7,8^, their usefulness in describing community structure is still a matter of debate^9–11^. While these co-occurrence studies are often performed on a relatively large number of individuals, they are limited to one or a few sampling points in time, presenting a mere snapshot of the dynamic microbiome. Other methods infer the ecological network by fitting an a priori chosen population-dynamic model to time series data of the microbial community^12–14^. These methods have the limitation that the inferred community structures strongly rely upon the assumptions that are intrinsic to the chosen model, and require considerable prior knowledge of the community of interest. There are also examples where the ecological interactions are inferred from repeated measurements around steady states^15^. This circumvents the need for a priori specification of a population dynamic model but makes the implicit assumption that the microbial system tends towards a stable equilibrium.

However, many experimental and field studies have shown the presence of complex dynamics in ecological communities, such as alternative stable states^16–18^ and oscillations and chaos^19–22^, questioning the steady states assumptions for the human microbiome. These dynamics are driven by a complex interplay between intrinsic factors (e.g., interaction mechanisms between organisms such as competition, mutualism and parasitism) and external perturbations (e.g., environmental conditions and interventions)^21,23,24^. Complex dynamics are also likely to occur in the human microbiome, because the bacterial communities living in our body are characterized by a plethora of interactions^25^ and are also affected by external perturbations (e.g., diet, use of antibiotics and travel patterns)^26–28^. A study with a thousand healthy western individuals suggested the existence of tipping elements in the intestinal microbiome^29^, reflecting the presence of alternative attractors and the possibility of more complex microbiome dynamics. The presence of complex dynamics in the human microbiome has not yet been demonstrated, probably due to the paucity of long and dense time series of the human microbiome. However, the study with one of the longest time series of human microbiome measurements available^30^ shows strong variability in the abundance of the bacteria over time, indicating that the human microbiome might not be at the presumed steady state.

To advance our ecological understanding of the human microbiome, methodology is needed that can exploit the temporal information in microbiome time series data without a priori knowledge of data generating mechanisms or steady-state assumptions. In the last decade, many methods have been developed to model the abundances of compositionally sampled data with the purpose of either fitting or predicting the temporal dynamics of the microbiota communities^31–33^. Here, we perform wavelet clustering analysis^34^, a technique that clusters time series based on similarities in their periodical patterns. This technique, which is commonly applied in climate and engineering studies^35^, more recently gained popularity in ecological^24^ and epidemiological^36–38^ studies. Wavelet clustering analysis has only recently been applied to time series derived from 16S amplicon data to reveal coastal plankton community structure^39^, but, to our knowledge, our study is the first application to human gut microbiome data. The novelty of the wavelet clustering approach, relative to prevailing co-occurrence or time-series methodologies in human microbiome research, is that it is able to characterize community structure on the basis of collective temporal behavior of the microbiota, without directly fitting a dynamic model or reconstructing the network of interacting species.

We illustrate wavelet clustering first with synthetic time series and then with densely sampled time series of human gut microbiome data from a male and female subject^30^. For both examples, we compare our results with clustering obtained on the basis of correlations in bacterial abundances over time. Our results show that correlation-based clustering is significantly different from clustering using wavelets. Wavelet clustering uncovered more diverse community structures and retained more of the differences between the male and the female subject compared to methods using temporal correlation. The results of this work highlight how the choice of method determines the type of communities found in microbiome data analysis. This is particularly important, considering that most of the putative microbiome communities, and their associations with a particular disease state or physical host condition, strongly rely on prevailing correlation-based methods or steady-state assumptions. Our results suggest that wavelet clustering readily capitalizes on the dynamic nature of the human microbiome and reveals more diverse community structures than those based on temporal correlations or associations.

## Results

### Wavelet cluster analysis

**Figure 1:**
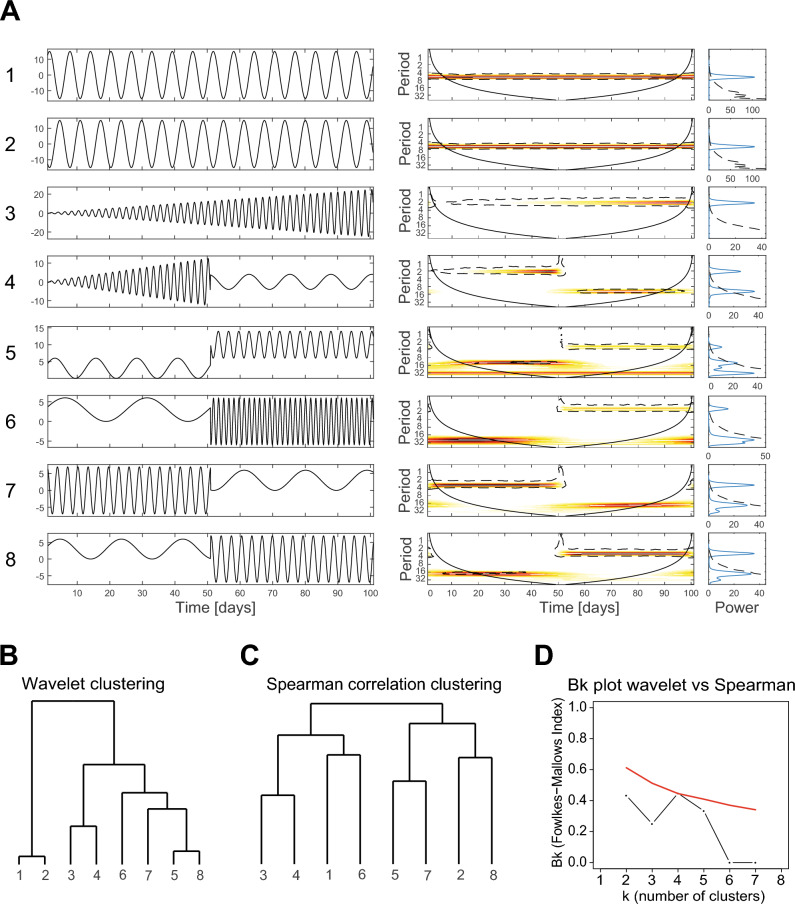
Illustration of wavelet clustering analysis with synthetic time series. (**A**) Wavelet analysis of synthetic time series: synthetic time series (left hand side) characterized by different periodicities; wavelet spectra (right hand side) and average wavelet spectra (far right) of the synthetic time series. Color codes represent wavelet power and range from low (white) to high (red). Black dotted lines enclose the 5% significance areas computed by using a Markov surrogate significance test. The solid black line delimits the cone of influence, where edge effects become important. (**B**,**C**) Clustering of the synthetic time series based on two methods: (**B**) Clustering based on the wavelet spectra. The cluster tree is constructed by grouping the time–frequency patterns of the time series using maximum covariance analysis. (**C**) Clustering based on Spearman correlations calculated for each pair of time series. The correlations are used to compute the dissimilarity matrix which is used to cluster the data. For both methods the hierarchical clustering of the time series is performed by using the WARD agglomeration criterion. (**D**) Comparison of the hierarchical clusters obtained using the *B*_*k*_ statistic^42^. Black dots represent the *B*_*k*_ values plotted against the *k* number of clusters in which each tree has been partitioned. Red line represents the one-sided rejection region based on the asymptotic distribution of *B*_*k*_ values, for each *k*, under the null hypothesis of no relation between the clusters (significance α = 5%).

Wavelet analysis enables investigation of time series characterized by different periodicities and is particularly suited for time series which are not stationary, as applies to many biological systems. We first illustrate this technique by using synthetic time series (Fig. 1A). Consider for instance time series 1 and 2: they are stationary and oscillate at the same periodicity of eight days, but in antiphase. They are therefore characterized by the same wavelet spectrum: a significant period of eight days (orange area inside the black dotted line) occurring along the entire time span of 100 days. The average wavelet spectrum, which is an estimation of the classical Fourier spectrum, is also identical among the two time series (see plot at the far most right-hand side). If one considers time series 7 and 8, one may see that they are showing opposite patterns. Time series 7 oscillates fast at a periodicity of about four days in the first 50 days and then slows down and oscillates at a periodicity of about 20 days in the second half of the time series. Time series 8 is doing exactly the opposite, it oscillates slowly with a periodicity of about 20 days in the first half of the time series and then oscillates with a periodicity of about four days in the second half of the time series. While the average wavelet spectrum is identical for both time series, the wavelet spectra are showing opposite patterns and are therefore able to depict the differences between the temporal behavior in the oscillations of the two time series (Fig. 1A).

The wavelet spectra are then compared using a procedure based on maximum covariance analysis^34^ which enables construction of a distance matrix based on the wavelet power spectra. The constructed distance matrix is used to build a cluster tree based on the WARD agglomeration criterion^40^ (Fig. 1B). For comparison, we also constructed a Spearman dissimilarity matrix calculated as *d* = 1 − *ρ* (where *ρ* is the correlation coefficient), using all data points in the time series pairs. The Spearman dissimilarity matrix is also used to construct a cluster tree based on the WARD agglomeration criterion (Fig. 1C). We compare the wavelet clustering with a clustering based on Spearman correlation, because the latter is a common method used in microbiome studies to infer relationships between microorganisms^41^. One may immediately observe substantial differences between the trees obtained with the two different methods (Fig. 1B,C). The time series are clustered differently within the trees according to the two methods, but also branching structure and the total length of the branches is noticeably different.

Time series 1 and 2 are close together in the wavelet cluster tree (Fig. 1B), but they fall apart in the Spearman cluster tree (Fig. 1C). The first results from the fact that the two time series have identical wavelet spectra, which indicates that the time series oscillate at the same periodicity. However, they are considered dissimilar in correlation analysis, because the time series are in antiphase (i.e., the peaks of one time series coincide with the troughs of the other time series and vice versa). Similarly, time series 5 and 8 cluster together in the wavelet tree but they fall apart in the Spearman cluster tree. Both time series 5 and 8 oscillate slowly at a periodicity of about 13 and 20 days respectively in the first part of the time series but then oscillate faster (at a periodicity of about four days) in the second part of the time series. Therefore, their wavelet spectra are very similar.

If the synthetic time series would represent the dynamical behavior of microorganisms, one would conclude from the Spearman cluster tree that microorganisms 1 and 2 (or 5 and 8) are not or only weakly related, because when one microorganism is highly abundant then the other one has very low abundance (and the other way around). The wavelet clustering instead shows that these microorganisms are strongly connected because they oscillate with similar periodicities and therefore share the same dynamical properties, which may point to ecological interdependence e.g. through parasitic interactions or neutral niche competition.

In addition to visual inspection, we used the *B*_*k*_ statistic^42^ to quantify the similarity in cluster trees constructed with the two methods. The *B*_*k*_ statistic assesses the chance-corrected proportion of items that two cluster trees have in common, as a function of the number of sub-clusters *k* that the two trees are partitioned into. Plotting *B*_*k*_ versus *k* gives a quantitative representation of the similarity between two cluster trees (black dots in Fig. 1D). The red line represents the 95% rejection region under the null hypothesis of no relation between the trees. For all partitions *k*, the blacks dots fall below the red line, hence we cannot conclude that the trees calculated with the wavelets and the Spearman correlations for the synthetic time series are significantly related.

In the Supplementary Information we give an additional demonstration of wavelet clustering analysis applied to the outputs of an ecological model of four consumers and four resources. In this case, wavelet clustering is able to correctly depict the competitive coupled dynamics between consumers and resources, whereas clustering based on Spearman correlation is not (Supplementary Fig. S1D,E).

### Application to human microbiome data

We tested our approach, as illustrated for the synthetic time series, on real data of microbiome communities. We used previously published gut microbiome time series of two healthy subjects, one male and one female, from whom fecal samples had been collected for 15 and 6 months respectively^30^. We considered the data at genus level and we selected the same 19 genera for the male and the female subject. A detailed description of the data and of the selection criterion is provided in the methods.

**Figure 2:**
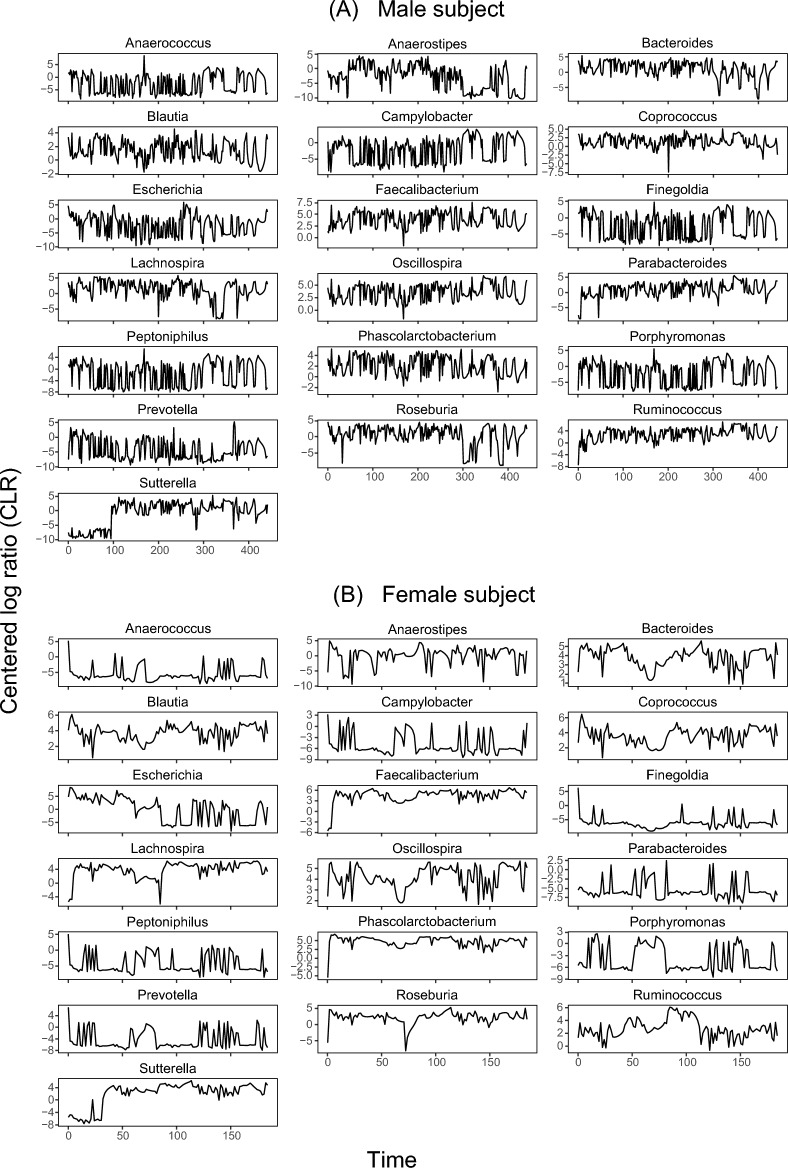
Gut microbiome time series of CLR transformed relative abundances for selected genera in the male (**A**) and female subject (**B**). The time series show clear fluctuations. Note the distinct time axes in the male and the female subject.

Time series of the selected genera for the male (upper panel) and the female subject (lower panel) are shown in Fig. 2. CLR transformed relative abundances over time show remarkable fluctuations. Some genera (e.g., *Lachnospira* and *Roseburia* in the male subject; *Bacteroides* in both subjects) show a clear wax and wane in their dynamical pattern. There are other genera (e.g., *Campylobacter* and *Finegoldia* in the female subject) that show more spiky dynamics, dominated by low CLR transformed relative abundances, but with few very high peaks.

**Figure 3:**
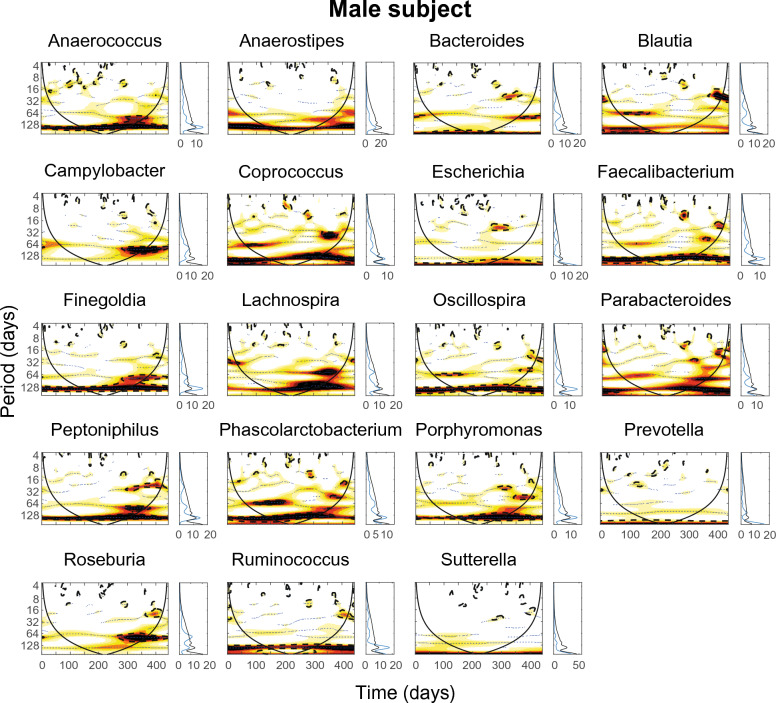
Wavelet analysis of the time series of selected genera for the male subject. For each genus the wavelet spectrum (left) and the average wavelet spectrum (right) are computed. Color codes represent wavelet power and range from low (white) to high (red). Black dotted lines enclose the 5% significance areas computed by using a Markov surrogate significance test. The solid black line delimits the cone of influence, where edge effects become important.

**Figure 4:**
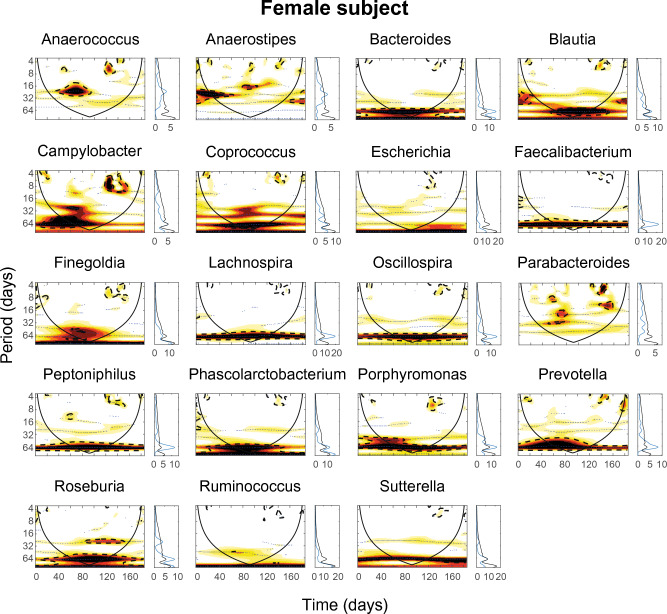
Wavelet analysis of the time series of selected genera for the female subject. For each genus the wavelet spectrum (left) and the average wavelet spectrum (right) are computed. Color codes represent wavelet power and range from low (white) to high (red). Black dotted lines enclose the 5% significance areas computed by using a Markov surrogate significance test. The solid black line delimits the cone of influence, where edge effects become important.

To capture possible similarities in the dynamical patterns of the bacteria, we applied wavelet analysis to each of the bacterial time series in both subjects. Wavelet spectra detected several significant periodicities in the fluctuations of bacteria both for the male (Fig. 3) and the female subject (Fig. 4). A first visual inspection of the spectra already reveals similarities between the dynamical patterns of the bacteria. For instance, in the male subject (Fig. 3), *Porphyromonas*, *Phascolarctobacterium* and *Peptoniphilus* show common periodicities of about 30–40 days co-occurring approximately for 100 days at the end of the timeseries. In addition, *Campylobacter* and *Roseburia* clearly show common periodicities of 64 days occurring approximately in the last 150 days of the timeseries, whereas *Blautia* and *Coprococcus* share this periodicity at the beginning of the timeseries. Common patterns are less clear in the female subject (Fig. 4), though some similar periodicities can be identified. For instance, many genera show the same periodicity of about 60 days occurring along the entire length of the time series.

**Figure 5:**
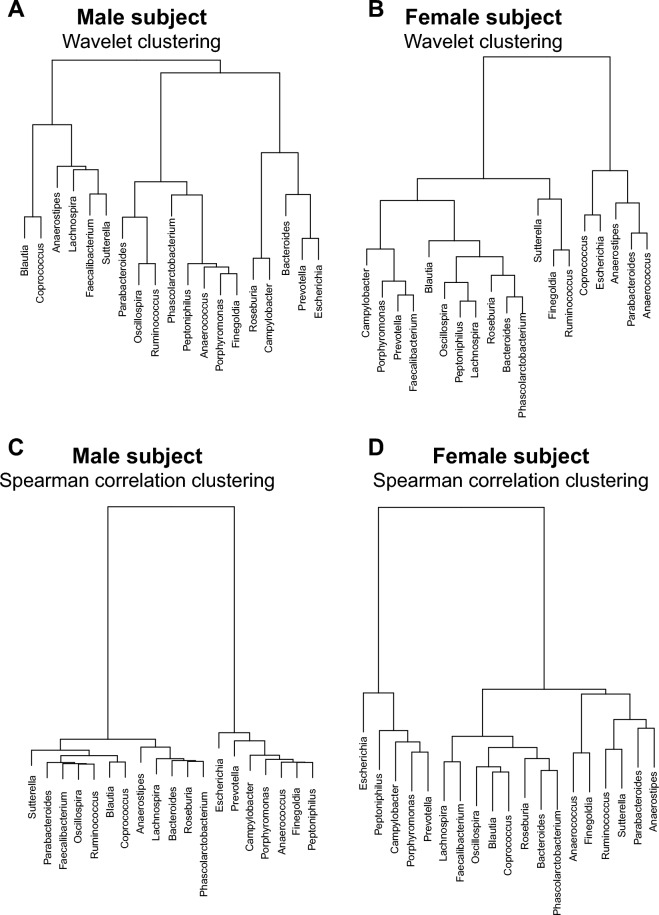
Clustering for the male and female subjects based on different methods. Cluster tree obtained by using the dissimilarity matrix obtained from the wavelet clustering analysis for (**A**) the male subject and (**B**) the female subject. Cluster tree obtained by using the dissimilarity matrix obtained from the Spearman correlation matrix for (**C**) the male subject and (**D**) the female subject.

With the wavelet spectra at hand, we built trees based on the wavelet distance matrix as described for the synthetic time series. Both the clusters based on wavelet spectra for the male and the female subject show a clear partition in two subgroups (Fig. 5A,B). The clusters based on Spearman correlations for the male and the female subjects are also characterized by two main sub clusters (Fig. 5C,D). Although there are few bacteria that are clustered together with both methods (i.e., *Peptoniphilus, Finegoldia, Porphyromonas *and* Anaerococcus* in the male subject), the two methods yield very different clusters. For instance *Bacteroides* and *Prevotella* are clustered together in the male subject with the wavelet method, but they are in two different clusters in the male subject with the correlation method. The case of *Prevotella* and *Bacteroides* resembles the example of signals 1 and 2 (or 5 and 8) illustrated above: two timeseries with similar dynamical properties are clustered together based on wavelets but are considered not related by the correlation method.

Also, visual comparison of the clusters obtained by using wavelets (Fig. 5A,B) with the clusters obtained by pairwise correlations (Fig. 5C,D) reveals substantial differences between the two methods in the positioning of branches within the two sub-clusters and in the total length of the branches. Of note, total branch length (see “Methods”) is substantially higher in the wavelet cluster tree as compared to the tree based on Spearman correlations (male subject: 80.9 vs 27.6; female subject: 70.0 vs 21.9). Further visual comparison of the trees based on wavelets among the two subjects also reveals that the members of each sub-cluster are substantially different between the male and the female subject (compare Fig. 5A with Fig. 5B). In contrast, comparison of the cluster trees based on correlations shows that many bacteria that are clustered together in the male subject are also clustered together in the female subject (compare Fig. 5C with Fig. 5D).

**Figure 6:**
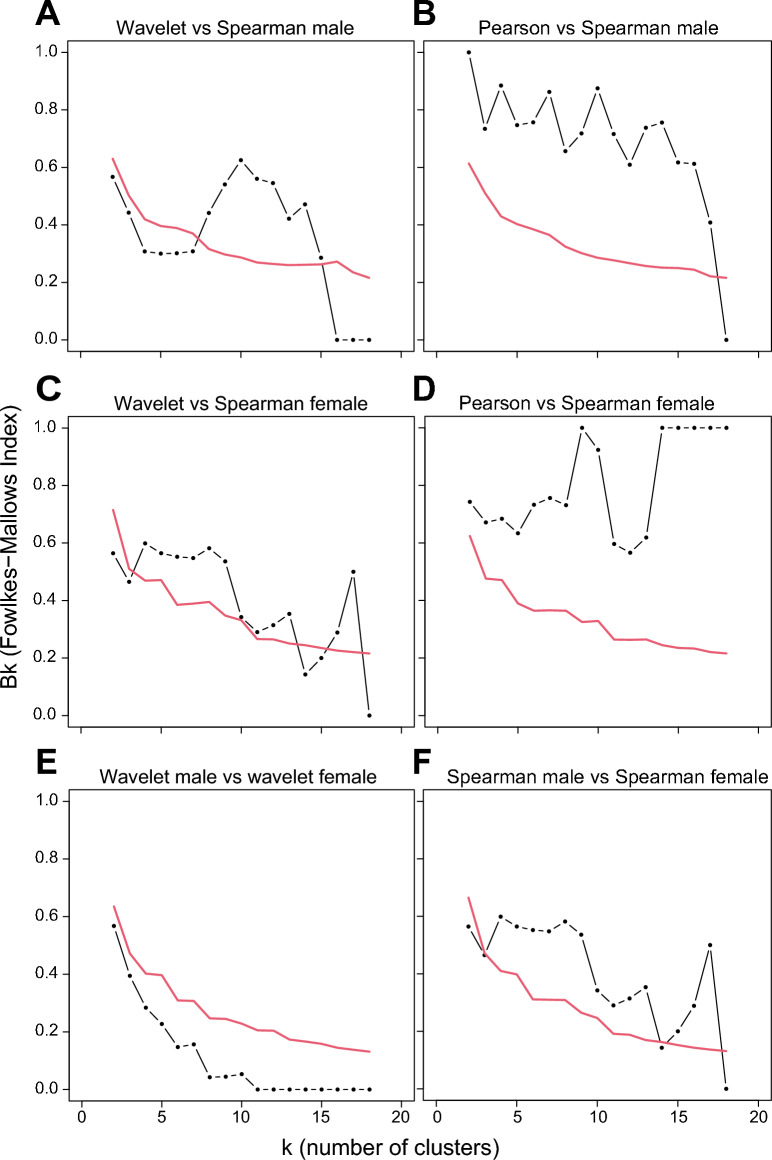
Comparison of hierarchical clusters using the *B*_*k*_ statistic^42^. Black dots represent the *B*_*k*_ values plotted against the *k* number of clusters in which the tree has been portioned. Red line represents the one-sided rejection region based on the asymptotic distribution of *B*_*k*_ values, for each *k*, under the null hypothesis of no relation between the clusters (significance α = 5%). (**A**) Comparison of the tree based on wavelets and the tree based on Spearman correlations for the male subject; (**B**) Comparison of the tree based on Pearson correlations and the tree based on Spearman correlations for the male subject; (**C**) Comparison of the tree based on wavelets and the tree based on Spearman correlations for the female subject; (**D**) Comparison of the tree based on Pearson correlations and the tree based on Spearman correlations for the female subject; (**E**) Comparison of the trees based on wavelets for the male and female subject; (**F**) Comparison of the trees based on Spearman correlations for the male and female subject.

To further quantify the similarities between subjects and methods we calculated the *B*_*k*_ statistic as we did for the synthetic time series. For low values of *k*, the dots in Fig. 6A,C fall below the 95% rejection line. Thus, wavelet clustering and Spearman clustering are not significantly related when the community is partitioned into a limited number of sub-clusters, and this holds for both the male and female subject. This is likely because the wavelet clustering method accounts for other features (i.e. the spectral characteristics of the bacterial dynamics and their time evolution) than the correlation-based methods, which only consider quantities averaged over the whole series. For higher values of *k*, the dots sometimes fall above the rejection line (Fig. 6A,C), meaning that wavelet clustering and Spearman clustering get significantly related at some higher resolution when certain sub-clusters become apparent. For comparison (Fig. 6B,D) we also applied the *B*_*k*_ statistic to correlation-based trees constructed with the Spearman correlation and with the Pearson correlation coefficient (trees not shown). For all *k* partitions (except the maximum partition in the male subject), the trees calculated with these two correlation methods are instead, as it could be expected, significantly related.

Finally, we also assessed the similarity between the two subjects. Interestingly, we found no evidence for related wavelet clusters between the male and female subjects, as all dots fall below the 95% rejection line irrespective the number of *k* partitions (Fig. 6E). In contrast, in the *B*_*k*_ plot of the Spearman correlation-based clustering the majority of dots fall above the 95% rejection line (Fig. 6F), indicating significantly related clusters for almost all sub-partitions between the male and female subject. This suggests that wavelet clustering not only uncovers more diverse community structures within individuals but might also be more sensitive towards subtle differences in community structures across individuals.

## Discussion

Developments in high-throughput sequencing have improved our ability to track the temporal variability of microbial communities. This has led to an increase in longitudinal data from a variety of different microbiomes ranging from wastewater^43^ to marine^44–46^ to freshwater^47^ to terrestrial^48,49^ environments. These time series offer unprecedented opportunities to gain ecological insights into microbial community dynamics and the mechanisms governing them, and to track the response of the microbial systems to external perturbations.

Ideally, long time series are required to capture the periodic patterns of microbial dynamics and reveal community structures. Unfortunately, only few of such datasets exists in human microbiome studies^26,30,50,51^. This probably reflects the relative difficulty to repeatedly sample the human microbiome in comparison to a natural field habitat (e.g., sampling strongly relies on the consent of the host to provide sampling material at a regular basis). As a result, the majority of studies on human microbial community structures have relied on sparse data and methods based on co-occurrence, which may have produced biased associations, e.g., towards positive correlations^41,52,53^. Clearly, there is a need to shift from a static to a dynamical approach, that takes into account the temporal development of bacterial communities and can shed new light on microbial community structure^54^. This also has bearing on the ability to employ microbiome data for clinical practice, as more and more studies move from association to prediction of disease course, e.g. exacerbation of Inflammatory Bowel Disease (IBD)^55^, and treatment response in *Clostridioides difficile* infection^56^.

Interestingly, our reanalysis of the widely used Caporaso et al*.* data reveals some novel important patterns. The trees obtained with the two different methods show significant differences in the way microbial genera are clustered together. For instance, there are cases where pairs of bacteria are clustered together in the male and female subject when using correlations, but not when using wavelets. For example, according to wavelet analysis *Blautia* and *Coprococcus* only cluster together in the male subject*,* and *Phascolarctobacterium, Roseburia* and *Bacteroides* only in the female subject, whereas these genera are clustered together in both subjects with the correlation-based method. In general, similarity of the cluster trees between subjects seems to be stronger with the correlation-based method than with wavelet clustering, for which we found no evidence for significant relations between the male and female trees. Tree correspondence according to clustering method within subjects was more ambiguous, as similarity also depends on tree resolution. This emphasizes how sensitive the clustering is to the type of method chosen.

In addition, we also note differences in the pattern of branching and in the total branch length of the cluster trees. Studies have shown that the total length of the branches in a traits tree is indicative of the functional diversity^57^ in ecosystems. Analogously, total branch length can here be considered as an indicator of community structure diversity. While we are not considering functional traits here, we could speculate that the higher total length observed in the wavelet clustering of the microbiome time series is indicative of a higher diversity in community structure as compared to the correlation-based method. A likely explanation is that wavelet analysis is able to detect dependencies that are not apparent in correlations, whereas the reverse is not the case: highly correlated time series are still detectable in wavelet spectra. Thus, wavelet clustering can extract more information on the dependencies within microbial communities than is reflected in mere correlations.

Looking at the clusters identified by the wavelet method one can speculate about possible interaction mechanisms between the bacteria. For instance, in the male subject, two genera are observed together, *Blautia* and *Coprococcus*. Members of genus *Blautia* are known to produce acetate and lactate which is shown to support improved growth of *Coprococcus* in vitro^58^. *Coprococcus* bacteria can convert lactate and acetate to butyrate, a short chain fatty acid that is associated with healthy microbiota^59^. This mutualistic mechanism could potentially lead to similar dynamical patterns and explain why these bacteria co-occur in the same cluster. Although these ‘potential’ interaction mechanisms are based on associative dynamical patterns of 16S rRNA gene abundance data they may provide ground for further investigation of these interactions in vitro and in vivo. In addition wavelet cluster analysis can be used as a starting point investigation for timeseries causality inference methods such as Granger causality^60,61^ or convergence-cross mapping^62,63^. For instance, there are methods that are able to estimate Granger’s causality from wavelet spectra of time series data^64,65^. Application to a complex system such as the microbiome has not yet been done and can be subject of investigation in future studies.

In ecological and epidemiological studies, wavelet analysis is often used to evaluate the effect of external factors, such as climatic or meteorological variables, on species or disease dynamics. Examples include studies which evaluate the effect of external factors on the spread of dengue fever^66^, malaria^67^ and cholera^68^ or on the dynamics of communities of benthic organisms^21^, marine^69^ and freshwater plankton^70^ or fish^24,71^. In an analogous way, when longitudinal studies on human microbiome dynamics become more widely available, metadata can be exploited using wavelet analysis to evaluate the effect of interventions, as for instance vaccination, the use of antimicrobials or probiotics, fecal microbiome transplantation and cancer treatment.

The reader interested in using the wavelet clustering approach might wonder how many points are needed for applying such analysis. The limits in the number of data points for wavelet analysis are similar to those of Fourier analysis and depend on the periodic components that one wants to highlight. For instance, Murdoch and colleagues^72^ suggest that with a minimum timeseries length of 25 time units one can identify periodicities between 2 time units (the Nyquist frequency) and 8–10 time units. Cazelles and colleagues^37^ are more conservative and they suggest timeseries with a minimum length of 30–40 time units which allows detection of a maximum periodicity equal to 20–25% of the total length of the timeseries. Another practical aspect is that wavelet analysis requires equidistant data. Although this might appear as a limiting factor, this requirement can easily be addressed. For instance, when possible, an experiment or a sampling strategy could be designed in such a way to obtain equidistant sampling points. If this is not possible, there are interpolation methods that can be used to obtain equidistant data. Different interpolation methods should be tested and the interpolated data should be checked against the original data to see if the general dynamical behavior is unaffected by the interpolation. This is the approach taken in this study. In addition, as for Fourier analysis, there are extensions of wavelet analysis that can be applied to non-equidistant data^73–77^.

In our study we analyzed the timeseries of two individuals and we compared the wavelet dendrograms of the two subjects by using a pairwise metric. Ideally, new longitudinal human microbiome studies will track the joint dynamics of much more than two individuals. When timeseries of multiple subjects become available, one might want to compare dendrograms among classes of individuals (e.g. individuals of the same gender or patients versus healthy controls). Instead of a pairwise metric between individuals, our analysis could then be applied to consensus dendrograms between classes of individuals^78^ to assess how communities differ with respect to the condition of interest.

To summarize, wavelet cluster analysis has the big advantage to account for non-stationary dynamics which are often preponderant in biological systems. In addition, we show that it appears to be a sensitive method for recovering microbial community structure from densely sampled microbiome time series. By taking into account the spectral features of bacterial abundance and their time evolution that are ignored in methods focusing on co-occurrence at any one time point, wavelet clustering analysis is able to extract more information on the dependencies within microbial communities, and to uncover more diverse communities within and across individuals than conventional methods. The results show that interpretation of microbial networks and communities, inferred on the basis of only a few sampling points in time, should be done with care, and be compared to alternatives.

## Methods

### Wavelet analysis

Wavelet analysis makes use of a periodic function (the mother-wavelet). The relative importance of periodicities (wavelet power) is then plotted in contour plots as a function of time (wavelet power spectra). Here we use as mother-wavelet the Morlet wavelet, which is particularly suited for detecting periodicities^35,79^. Significance of the detected periodicities is assessed by using a Markov surrogate significance test^80^. Statistical significance is assessed by testing against the null hypothesis that observed periodicities are identical to those generated by a stochastic Markov process, characterized by the same mean, the same variance, the same distribution of values and the same short-term autocorrelation structure. More detailed information on wavelet analysis is provided elsewhere^21,81–83^.

### Wavelet clustering

The wavelet spectra are compared using a procedure based on the maximum covariance analysis^34^.

To be more precise, as described in Fromentin et al.^34^, the distance matrix is computed based on leading patterns and singular vectors obtained using matrix decomposition analysis. Matrix decomposition analysis relies on a singular value decomposition performed on the covariance matrix between two wavelet power spectra. This enables construction of a distance matrix based on the wavelet power spectra. Only periodicities with a confidence higher than 90% have been considered in the computation of the dissimilarity matrix. Wavelet analysis and wavelet clustering were performed by using wavelet software written in Matlab^34^ which is available at (https://www.biologie.ens.fr/~cazelles/bernard/Research.html).

### Comparison among cluster trees

We quantified similarities between cluster trees by using the *B*_*k*_ statistic. The *B*_*k*_ statistic measures the degree of similarity between two hierarchical clusters. Consider two hierarchical trees *C*_*1*_ and *C*_*2*_, each with the same number of elements *n* and partition each tree to produce *k* = 2, …, *n*—1 sub-clusters for each tree. For each value of* k* we can compute the quantity *m*_*i,j*_ which quantifies the number of objects in common between the *i*th cluster of *C*_*1*_, and the *j*th cluster of *C*_*2*_.

The statistic *B*_*k*_ is then defined:

1 $$B_{k} = \frac{{T_{k} }}{{\sqrt {P_{k} Q_{k} } }}$$

where:

2 $$T_{k} = \sum\limits_{i = 1}^{k} {\sum\limits_{j = 1}^{k} {m_{i,j}^{2} - n;} }$$

3 $$P_{k} = \sum\limits_{i = 1}^{k} {\left( {\sum\limits_{j = 1}^{k} {m_{i,j} } } \right)}^{2} - n;$$

4 $$Q_{k} = \sum\limits_{j = 1}^{k} {\left( {\sum\limits_{i = 1}^{k} {m_{i,j} } } \right)^{2} } - n;$$

*B*_k_ is calculated for all the *k* partitions and *B*_*k*_ takes values between 0 and 1; *B*_*k*_ = 1 indicates that *k* sub-clusters in each tree correspond completely whereas *B*_*k*_ = 0 indicates that the sub-clusters in each tree don’t correspond at all. Details on the *B*_*k*_ statistic are described in^42^. The *B*_k_ statistic has been calculated by using the R package “Dendextend”^84^. The computed values of *B*_*k*_ are then plotted as a function of *k*. The significance of the *B*_*k*_ values is tested against the null hypothesis that the two cluster trees are not related. A one-sided rejection line (with significance level of 5%) is drawn based on the asymptotic distribution of *B*_*k*_ values, for each *k*, under the null hypothesis of no relation between the clusters.

### Calculation of total branch length

The total branch length was calculated by summing the lengths of connecting segments in the tree by using the function *treeheight* of the Rpackage “Vegan”^85^.

### Microbiome data

In our analysis, we used previously published time series of the gut microbiome of two healthy subjects, one male and one female, on which fecal samples have been taken for 15 and 6 months respectively^30^. The V4 variable region of the 16S rRNA gene was amplified by PCR and sequenced on an Illumina Genome Analyzer IIx. In the original paper of Caporaso et al.^30^ the raw sequences were clustered in Operational Taxonomic Units (OTU) using the Quantitative Insights Into Microbial Ecology (QIIME) pipeline. However, recent studies have shown that the use of OTUs is more prone to produce noisy features which are artifacts of sequencing errors^86^. Nowadays, the use of Amplicon Sequence Variants (ASV) data has been shown to be more reliable than OTU’s^86^.

Following the same line, here we used the ASV gut microbiome data of Caporaso et al.^30^ which is available at the Earth Microbiome Project (EMP) platform (https://earthmicrobiome.org/). The ASV data provided at the EMP platform have been generated from the raw sequence data with the Deblur pipeline^87^ and the detailed protocol is provided in^88^. The data for human microbiome time series was obtained from “emp_deblur_150bp.release1.biom” by filtering to keep only samples from the Qiita study ID 5501.

We removed singletons and ASV sequences assigned to mitochondria and chloroplasts. We assembled the taxa at the genus level and this yielded 578 unique genera. For both the male and female subject, we first removed samples with less than 500 reads, then we transformed the time series to relative abundances and then we made a selection of genera, by using a bootstrapping method^89^ with a prevalence value of 25% and a relative abundance threshold value of 0.005 (i.e. select the genera in which the relative abundance has a value higher than 0.005 in at least 25% of the samples). We disregarded the taxa that were not identified as uniquely defined genera. This yielded a total of 19 genera for the male subject and of 12 genera for the female subject. The aim of our analysis is to compare clusters (and techniques to obtain these clusters) among the two different subjects. Therefore, we considered in our analysis the genera that were present in at least one subject, yielding a total of 19 genera for each subject. Processing of the data from ASV to the core-microbiome taxa was done by using the R-packages phyloseq^90^ and microbiome^89^. Subsequently, we applied a CLR (centered log ratio) transformation to the relative abundance timeseries by using the Rpackage “compositions”. The CLR transformed timeseries of the selected genera are shown in Fig. 2. Wavelet analysis requires equidistance between subsequent datapoints, therefore we interpolated the time series of both subjects using cubic hermite interpolation to obtain data with equidistant time intervals of 1,6 days (the mean time interval of the original data of the male subject is 1.6 days and the female subject is 1.5 days), yielding a total of 336 data points for the male subject and of 131 data points for the female subject.

Before performing wavelet analysis to the data, the microbiome CLR transformed time series were rescaled by using a Box–Cox transformation to suppress sharp peaks, homogenize the variance and approximate a normal distribution. For each timeseries the optimal parameter of the Box–Cox transformation has been estimated by optimizing the normal probability plot correlation coefficient by using the Rpackage “envstats”(see Supplementary Fig. S2 in the Supplementary Information).

## Supplementary Information

Supplementary Figure S1.

Supplementary Figure S2.

Supplementary Information.

## Data Availability

The datasets analyzed during the current study are from Caporaso et al.^30^ and are publicly available at https://earthmicrobiome.org/.
