# Supplementary material for: Wavelet clustering analysis as a tool for characterizing community structure in the human microbiome
Source: Sci Rep. 2023 May 17;13:8042. doi: 10.1038/s41598-023-34713-8 (PMC10192422; doi:10.1038/s41598-023-34713-8)
Supplement: Supplementary file 1 — Supplementary Figure S1. [file 41598_2023_34713_MOESM1_ESM.pdf]

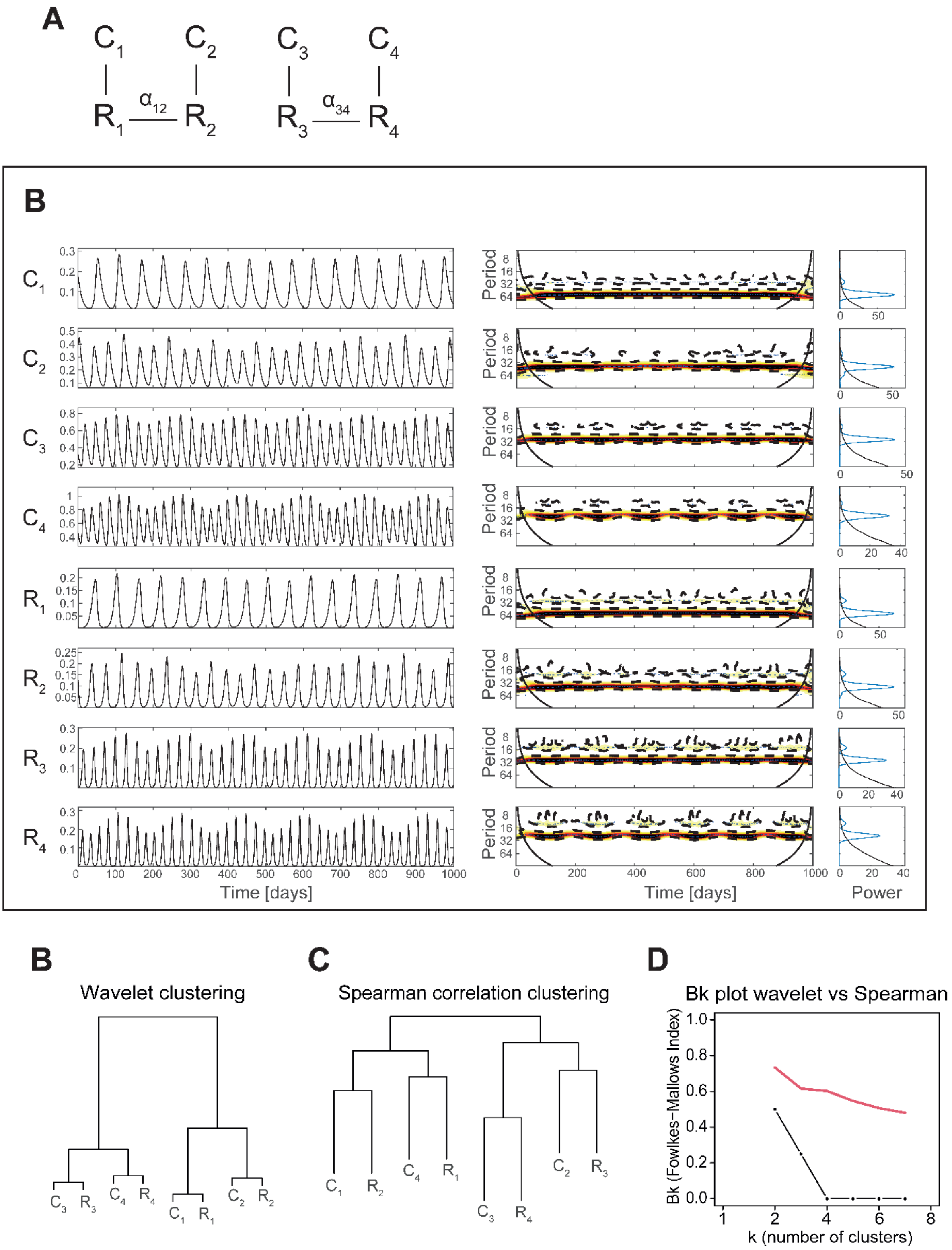
A
B
C
D
Time [days]
Period
Power
Wavelet clustering
Spearman correlation clustering
Bk plot wavelet vs Spearman
Bk (Fowlkes-Mallows Index)
k (number of clusters)

Supplementary Figure S1 Application of wavelet clustering to the outputs of a model with four consumers feeding on four resources. **A,** The model consists of two separated food webs of two consumers on two resources. The two resources within each food web negatively interact with a competition coefficient α. **B,** Outputs of the resources-consumers model. Simulations have been run for 2000 time units .The plots shown here covers the last 1000 time units of the simulation. Parameters: K = 1; a = 2; b = 1.3; m = 0.1; r_1=0.2; r_2 = 0.4; r_3=0.8; r_4=1.2;α_{12} = 0.8; α_{34} = 0.4; **C,** Wavelet spectra (right hand side) and average wavelet spectra (far right) of the model outputs. Color codes represent wavelet power and range from low (white) to high (red). Black dotted lines enclosed the 5% significance areas computed by using a Markov surrogate significance test. The solid black line delimits the cone of influence, where edge effects become important. Clustering based on the wavelet spectra. The cluster tree is constructed by grouping the time–frequency patterns of the time series using maximum covariance analysis. **D**, Clustering based on Spearman correlations calculated for each pair of time series. The correlations are used to compute the dissimilarity matrix which is used to cluster the data. For both methods the hierarchical clustering of the time series is performed by using the WARD agglomeration criterion. **E**, Comparison of the hierarchical clusters obtained using the B_k statistics. Black dots represent the B_k values plotted against the *k* number of clusters in which each tree has been partitioned. Red line represents the one-sided rejection region based on the asymptotic distribution of B_k values, for each *k*, under the null hypothesis of no relation between the clusters (significance α =5%).
