# Supplementary material for: Wavelet clustering analysis as a tool for characterizing community structure in the human microbiome
Source: Sci Rep. 2023 May 17;13:8042. doi: 10.1038/s41598-023-34713-8 (PMC10192422; doi:10.1038/s41598-023-34713-8)
Supplement: Supplementary file 2 — Supplementary Figure S2. [file 41598_2023_34713_MOESM2_ESM.pdf]

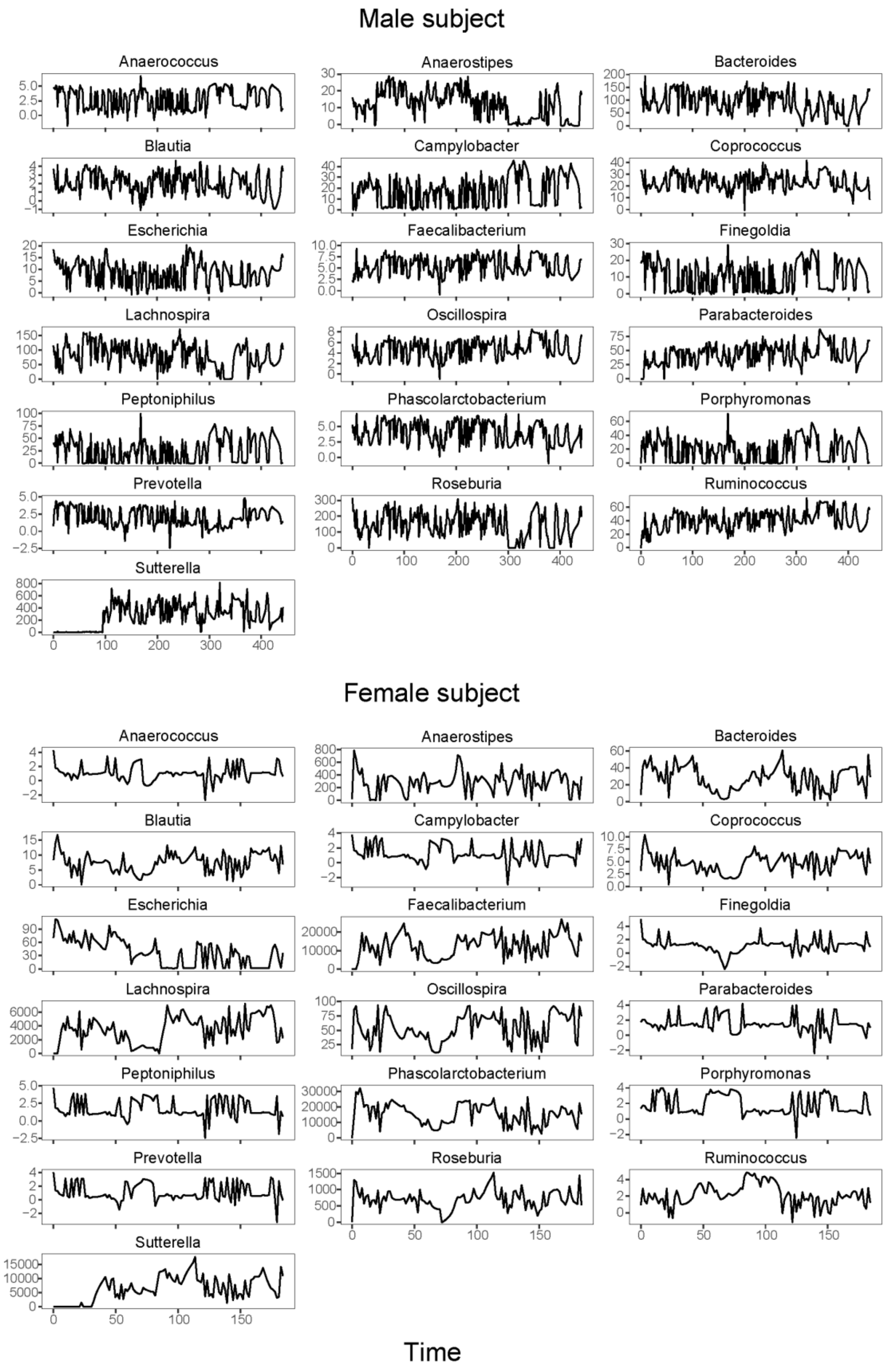

Supplementary Figure S2 Box-cox transformed CLR time series of selected genera in the male, **A** and female subject, **B**. The relative abundance time series of both subjects have been interpolated

using cubic Hermite interpolation to obtain data with equidistant time intervals of 1,6 days (the mean time interval of the original data of the male subject is 1.6 days and the female subject is 1.5 days), yielding a total of 336 data points for the male subject and of 131 data points for the female subject. Subsequently, we applied a CLR (centered log ratio) transformation to the relative abundance timeseries by using the Rpackage “compositions”. Before performing wavelet analysis to the data, the microbiome CLR transformed time series were rescaled by using a Box-Cox transformation to suppress sharp peaks, homogenize the variance and approximate a normal distribution. For each timeseries the optimal parameter of the Box-Cox transformation has been estimated by optimizing the normal probability plot correlation coefficient by using the Rpackage “envstats”.
