# Supplementary material for: Wavelet clustering analysis as a tool for characterizing community structure in the human microbiome
Source: Sci Rep. 2023 May 17;13:8042. doi: 10.1038/s41598-023-34713-8 (PMC10192422; doi:10.1038/s41598-023-34713-8)
Supplement: Supplementary file 3 — Supplementary Information. [file 41598_2023_34713_MOESM3_ESM.pdf]

Supplementary Information: Wavelet clustering analysis as a tool for characterizing community structure in the human microbiome

Elisa Benincà, Susanne Pinto, Bernard Cazelles, Susana Fuentes, Sudarshan Shetty, and Johannes A. Bogaards

Wavelet clustering applied to the dynamics of four consumers feeding on four resources.

In this section we give an extra demonstration of the potential of wavelet clustering by performing the analysis on the outputs of a simplified ecological model describing the dynamics of four consumers and four resources. The model is a modified version of the previously published model of Vandermeer[1] (see also[2,3]) of two species feeding on two resources.

The model reads as follows:

$$\frac{dC_i}{dt} = \frac{aR_iC_i}{1+bR_i} - mC_i;$$

$$\frac{dR_i}{dt} = r_iR_i\left(\frac{K - R_i - \alpha_{ij}R_j}{K}\right) - aR_i\left(\frac{C_i}{1+bR_i}\right);$$

$$\frac{dC_k}{dt} = \frac{aR_kC_k}{1+bR_k} - mC_k$$

$$\frac{dR_k}{dt} = r_kR_k\left(\frac{K - R_k - \alpha_{kl}R_l}{K}\right) - aR_k\left(\frac{C_k}{1+bR_k}\right);$$

for i = 1, 2 and k= 3, 4 and $i \neq j$ and $k \neq l$, where C_i and C_k are the abundances/densities of the ith and the kth consumers respectively, and R_i and R_k denote those of the ith and the kth resources. The parameters r_i and r_k are the intrinsic growth rates of the ith and the kth resource respectively, m is the mortality rate of the consumers, α_{ij} is the competition coefficient between resource 1 and 2, α_{kl} is the competition coefficient between resource 3 and 4, a is the resource consumption rate, b is the functional response parameter (with higher values denoting diminished response in consumer growth at a given resource abundance) and K is the carrying capacity of each resource, which we assume for simplicity to be the same for all four resources.

The model consists of two separated food webs of two consumers each feeding on one resource (Supplementary Fig. S1A). Consumer C_1 feeds on resource R_1, consumer C_2 feeds on resource R_2 and the two resources R_1 and R_2 negatively interact with a parameter α_{12}. Similarly consumer C_3 feeds on resource R_3, consumer C_4 feeds on consumer R_4 and the two resources R_3 and $R4$ negatively interact

with a parameter α_{34}. In figure S1B are shown the temporal dynamics of the four consumers and the four resources. We applied wavelet analysis to all eight of the timeseries (Supplementary Fig. S1C) and we used this information to build the cluster tree (Supplementary Fig. S1D). Wavelet clustering identifies two big sub-clusters: sub-cluster 1 with consumers C_3 and C_4 and resources R_3 and R_4 and sub-cluster 2 with consumers C_1 and C_2 and resources R_1 and R_2. Wavelet clustering is able to identify the two separated food webs. In addition inside each cluster we observe that each consumer is clustered together with its own resource (C_1 with $R1$, C_2 with R_2, C_3 with R_3, C_4 with R_4). For comparison we build a tree based on Spearman correlation (Supplementary Fig. S1E). In contrast to wavelet clustering, clustering based on Spearman correlation is not able to identify neither the two distinct food webs, neither the pairs of consumers-resources. Clustering based on Spearman correlation is substantially different from clustering based on wavelets as it is shown by the corresponding B_k plot (Supplementary Fig. S1F).

References

1 Vandermeer, J. Coupled oscillations in food webs: balancing competition and mutualism in simple ecological models. *The American Naturalist* **163**, 857-867 (2004).

2 Benincà, E., Jöhnk, K. D., Heerkloss, R. & Huisman, J. Coupled predator–prey oscillations in a chaotic food web. *Ecology letters* **12**, 1367-1378 (2009).

3 Massoud, E. C. *et al.* Probing the limits of predictability: data assimilation of chaotic dynamics in complex food webs. *Ecology letters* **21**, 93-103 (2018).
